# Secondary Neuropathy From Polyneuropathy, Organomegaly, Endocrinopathy, Monoclonal Gammopathy, and Skin Changes (POEMS) Syndrome: A Case Report

**DOI:** 10.7759/cureus.95064

**Published:** 2025-10-21

**Authors:** Peter Young, Maxwell Kroloff

**Affiliations:** 1 Internal Medicine/General Internal Medicine and Health Services Research, University of California, Los Angeles, Los Angeles, USA; 2 Oncology, University of California, Los Angeles, Los Angeles, USA

**Keywords:** acquired neuropathy, distal symmetric polyneuropathy, peripheral neuropathy, plasma cell disorder, poems polyneuropathy, poems syndrome, polyneuropathy, secondary neuropathy

## Abstract

We report the case of a 62-year-old woman who presented with signs and symptoms of distal sensory polyneuropathy. Her initial electromyography and nerve conduction studies (EMG/NCS) showed a severe length-dependent neuropathy with uniform demyelination and secondary axonal changes. After extensive evaluation for secondary causes, she was ultimately found to have polyneuropathy, organomegaly, endocrinopathy, monoclonal gammopathy, and skin changes (POEMS) syndrome. She underwent treatment with lenalidomide and dexamethasone, followed by an autologous stem cell transplant, and is now doing well with marked improvement in her function. This case emphasizes the importance of evaluating for secondary causes of polyneuropathy and provides a brief overview of POEMS syndrome.

## Introduction

Distal symmetric polyneuropathy (DSP) is the most common subtype of peripheral neuropathy [1]. It can be idiopathic but is often associated with secondary causes [2]. These can include diabetes mellitus, nutritional deficiencies, and toxins such as alcohol or chemotherapy, as well as infections such as human immunodeficiency virus (HIV) or inflammatory conditions [2]. One rare cause of DSP is a paraneoplastic condition characterized by polyneuropathy, organomegaly, endocrinopathy, monoclonal gammopathy, and skin changes, otherwise known as POEMS syndrome [3]. POEMS syndrome is a rare, multi-system disorder driven by a plasma cell clone that can present with a rapidly progressive symmetric ascending polyneuropathy but often involves other debilitating end-organ manifestations as well [4]. Early diagnosis is crucial to prevent progressive functional decline and life-threatening complications [4].

A diagnosis of POEMS requires the presence of peripheral neuropathy and a monoclonal plasma cell disorder, as well as one major criterion and one minor criterion [3]. Major criteria include osteosclerotic or mixed sclerotic/lytic lesions on imaging, Castleman disease, and elevated plasma vascular endothelial growth factor (VEGF) levels [3]. Minor criteria include organomegaly, volume overload, endocrinopathy, skin changes, papilledema, and thrombocytosis or polycythemia [3]. Given its rarity and poorly understood pathogenesis, there is no standardized treatment for POEMS, though most protocols target what is thought to be an underlying clonal plasma cell process.

## Case presentation

A 62-year-old woman presented to the clinic with two years of progressive sensitivity and heaviness in her lower extremities. She was experiencing intermittent swelling in her feet that extended up to her calves, as well as numbness, tingling, and burning pain particularly at night and a sensation of tightness in her skin. She reported thinner and more wrinkled skin with acneiform changes on her face. She had become dependent on a cane for ambulation and had suffered multiple falls due to her symptoms. She also endorsed weight loss of at least 40 pounds over 12 months and an enlarged thyroid. She had no fevers, chills, night sweats, lymphadenopathy, back pain, or trauma. She had a history of transient ischemic attack and was on aspirin 81 mg daily and took gabapentin 300 mg three times a day for neuropathic pain. Past medical, family, and social histories were otherwise noncontributory.

At her initial visit, her vitals were unremarkable. Physical exam was notable for 1+ pitting edema to her calves bilaterally. She had significantly diminished pinprick and proprioceptive sensation in her lower extremities, with mildly reduced sensation in her upper extremities. Motor testing revealed 3/5 strength in dorsiflexion and plantarflexion bilaterally, 3+/5 strength in knee flexion and extension, and 4/5 strength in her hips. Upper extremity strength was 4/5 throughout, and she had absent reflexes at the patella, Achilles, biceps, and brachioradialis tendons bilaterally with a downward plantar response. Coordination was intact, but gait was markedly unsteady.

Labs (Table 1) were notable for a monoclonal IgA lambda protein on serum immunofixation (IFE) but normal serum protein electrophoresis (SPEP), urine protein electrophoresis (UPEP), and serum free light chains. Comprehensive metabolic panel, complete blood count, and urinalysis were normal. She had a negative HIV and rapid plasma reagin (RPR) test; normal B12, B6, and B1 levels; and normal thyroid-stimulating hormone (TSH). Magnetic resonance imaging (MRI) of her lumbar and cervical spine was performed, which showed degenerative changes and foraminal narrowing but no cord signal abnormality. Electromyography and nerve conduction studies (EMG/NCS) revealed a very severe length-dependent neuropathy with uniform demyelination and secondary axonal changes, with more dramatic findings in the lower versus upper extremities.

**Table 1:** Summary of Lab Results Relevant lab results from the patient's evaluation are summarized here with reference ranges. Serologic workup for secondary causes was unremarkable until VEGF levels returned significantly elevated, suggestive of POEMS syndrome. HIV: human immunodeficiency virus; RPR: rapid plasma reagin; TSH: thyroid-stimulating hormone; SPEP: serum protein electrophoresis; IFE: immunofixation; VEGF: vascular endothelial growth factor; mcIU/mL: milli-international units per milliliter; nmol/L: nanomole per liter; nmol/mL: nanomole per milliliter; pg/mL: picogram per milliliter; mg/dL: milligram per deciliter.

Lab test	Lab value	Reference range
HIV antigen/antibody test	Nonreactive	Nonreactive
RPR	Nonreactive	Nonreactive
TSH	0.62 mcIU/mL	0.3-4.7 mcIU/mL
Vitamin B1	105 nmol/L	70-180 nmol/L
Vitamin B6	20.6 nmol/mL	20-125.0 nmol/mL
Vitamin B12	380 pg/mL	254-1,080 pg/mL
Hemoglobin A1c	5.4%	<5.7%
SPEP	No monoclonal bands detected	
Serum IFE	Monoclonal IgA lambda protein present	
Kappa/lambda light chain ratio	1.18	0.26-1.65
C-reactive protein	0.5 mg/dL	<0.8 mg/dL
VEGF	1,417 pg/mL	31-86 pg/mL

**Figure 1:**
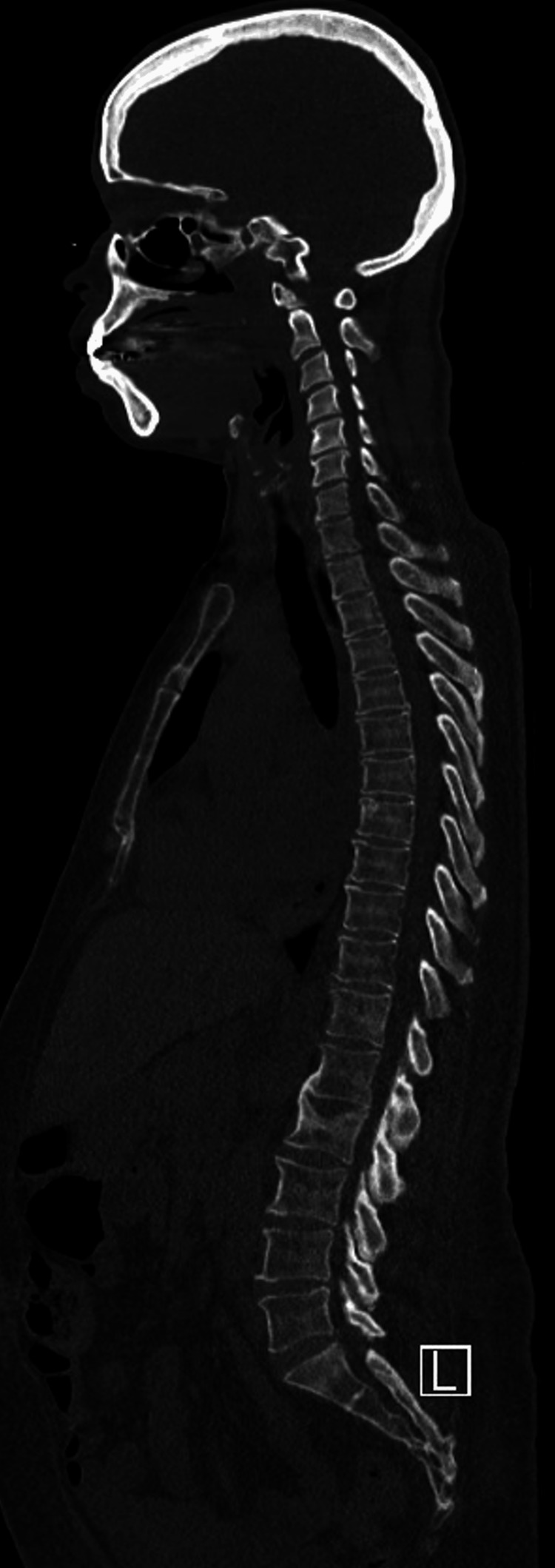
Normal CT Myeloma Survey Sagittal section of the CT myeloma survey, which did not reveal any lytic lesions and was overall unremarkable. Osteosclerotic or mixed sclerotic/lytic lesions are one of the three major criteria for POEMS. In this case, the patient satisfied a major criterion by the presence of elevated plasma VEGF protein. CT: computed tomography; POEMS: polyneuropathy, organomegaly, endocrinopathy, monoclonal gammopathy, and skin changes; VEGF: vascular endothelial growth factor

**Figure 2:**
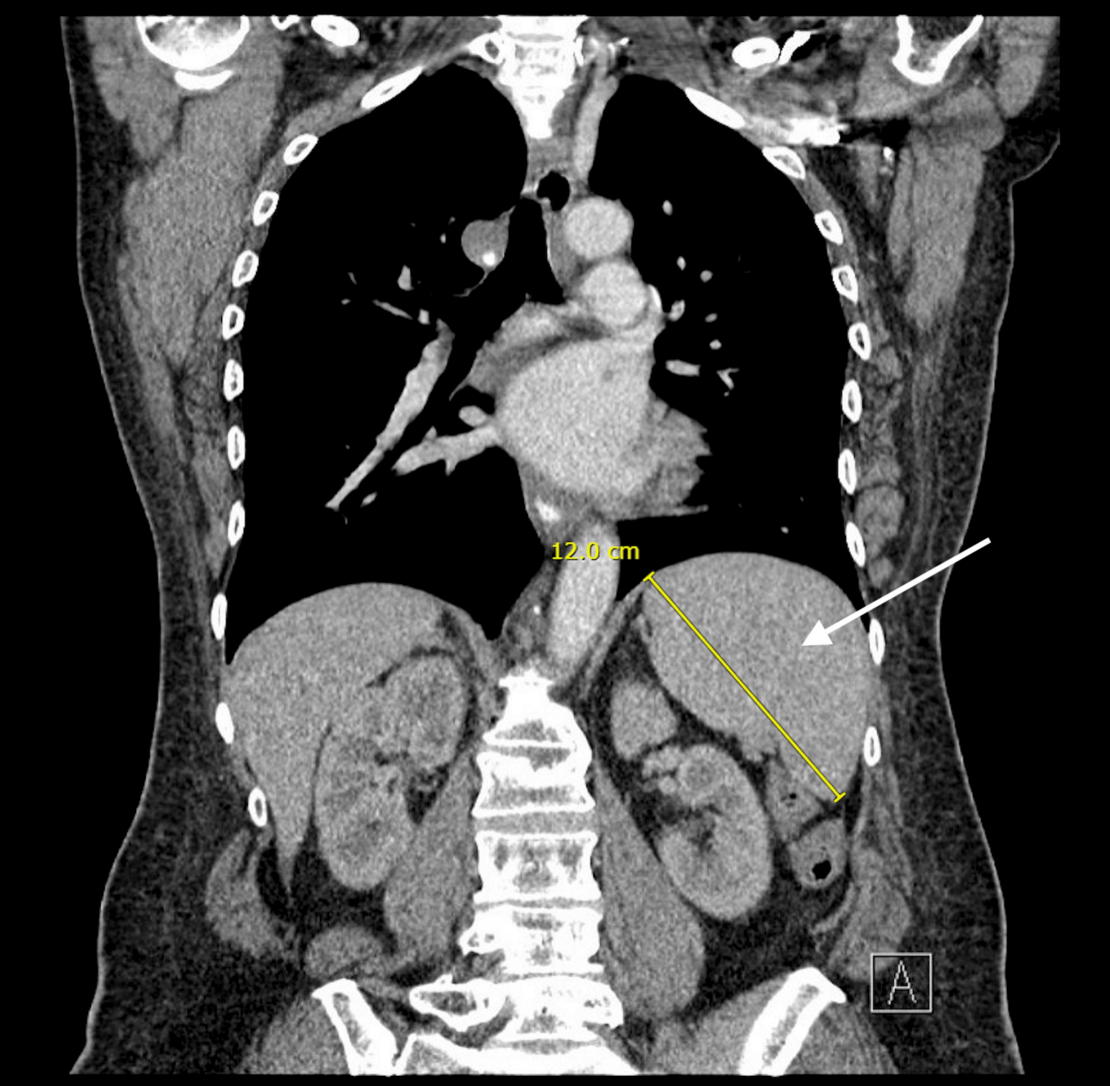
CT Scan Showing Borderline Splenomegaly CT abdomen/pelvis showing borderline splenomegaly of approximately 12 cm, a nonspecific finding but also one of the minor criteria for POEMS syndrome (organomegaly). CT: computed tomography; POEMS: polyneuropathy, organomegaly, endocrinopathy, monoclonal gammopathy, and skin changes.

**Figure 3:**
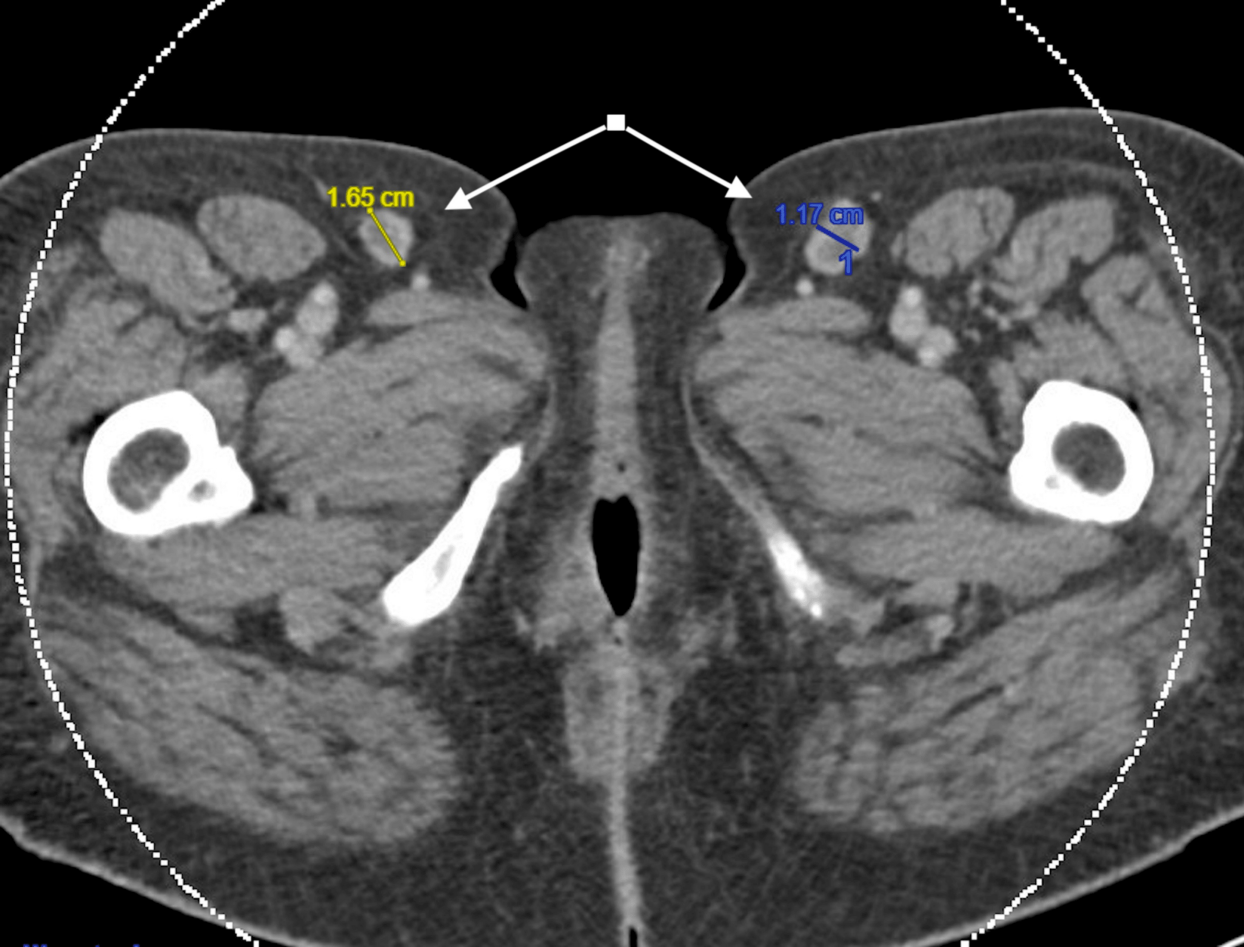
CT Scan Showing Inguinal Lymphadenopathy CT abdomen/pelvis revealed mild lymphadenopathy, most notable in the inguinal region. Lymphadenopathy qualifies as organomegaly, which is one of the minor criteria for POEMS syndrome. Other minor criteria include extravascular volume overload, endocrinopathy, skin changes, papilledema, and thrombocytosis or polycythemia. CT: computed tomography; POEMS: polyneuropathy, organomegaly, endocrinopathy, monoclonal gammopathy, and skin changes.

She was seen urgently by neurology and hematology within the next three weeks. A myeloma skeletal survey did not reveal any lytic lesions (Figure 1), and a computed tomography (CT) scan of her chest, abdomen, and pelvis showed mild widespread lymphadenopathy along with borderline splenomegaly (Figures 2, 3). Bone marrow biopsy was normal but showed a small population of 7%-8% lambda-restricted plasma cells, consistent with plasma cell dyscrasia. Based on the presence of a severe peripheral neuropathy as well as a monoclonal plasma cell disorder, there was concern for potential POEMS syndrome. To investigate further, VEGF levels were sent and returned at 1,412 pg/mL (with an upper limit of normal being 86). She was diagnosed with POEMS and started on lenalidomide and dexamethasone. Six months later, she underwent an autologous stem cell transplant. Daratumumab induction had been planned but was denied by insurance despite multiple appeals. With this treatment and aggressive rehabilitation, she has had significant improvement in function, and one year later, she is now ambulatory without any assistive devices. She has remained off chemotherapeutics with close surveillance of her neuropathic symptoms and lab work including SPEP/IFE, kappa/lambda light chains, and VEGF levels.

## Discussion

This patient presented with symmetric sensory symptoms along with muscle weakness predominantly affecting her distal extremities, consistent with a DSP. Evaluating for secondary causes of neuropathy is a crucial part of the initial workup. There are numerous etiologies that contribute to DSP, which is the most common subtype of neuropathy. Diabetes mellitus is the most common cause (18%-49% of cases), followed by alcohol use, toxins and chemotherapeutic drugs, nutritional deficiencies, immune-mediated causes, and hereditary conditions (most commonly Charcot-Marie-Tooth disease) [1]. Chronic idiopathic axonal polyneuropathy (CIAP) is a diagnosis of exclusion, which is responsible for between 12% and 49% of cases [2].

This patient was ultimately found to have POEMS syndrome, a rare paraneoplastic syndrome characterized by polyneuropathy, organomegaly, endocrinopathy, monoclonal gammopathy, and skin changes [3]. The pathogenesis is not fully understood but thought to be related to increased VEGF among other proinflammatory cytokines driven by a clonal plasma cell process [5]. As in the above patient, a rapidly worsening, symmetric ascending peripheral polyneuropathy is often the initial presenting feature, emphasizing the importance of early diagnosis and intervention [4]. Yet, distinguishing POEMS from alternative etiologies of peripheral neuropathy such as amyloid light chain (AL) amyloidosis or chronic inflammatory demyelinating polyneuropathy (CIDP) can be challenging.

To avoid lasting end-organ manifestations, clinicians must maintain a high index of suspicion for POEMS, especially in the setting of a symmetric length-dependent peripheral neuropathy and a monoclonal gammopathy. NCS can show demyelination similar to CIDP but has distinguishing characteristics including more severe axonal loss [6]. Aside from the neuropathy, the prevalence of other manifestations varies greatly. In addition to a complete history and physical exam, complete blood count, and comprehensive metabolic panel, the diagnostic workup should include a serum and 24-hour urine collection with electrophoresis and IFE, as well as quantitative immunoglobulins and a serum free light chain assay. A serum VEGF level is also indicated, in addition to hormonal studies, to screen for endocrinopathy. In terms of imaging, a CT survey or PET-CT is necessary to evaluate for osteosclerotic lesions, as a skeletal survey is likely to miss occult lesions. An echocardiogram should be performed to assess right-sided pressures, and finally, a bone marrow aspirate and biopsy with fluorescence in situ hybridization (FISH) panel for myeloma should be completed. Interestingly, the bone marrow plasma cell clone is often a relatively small size (~5% in 50% of patients) with POEMS [5]. Mandatory criteria for the diagnosis of POEMS syndrome require a polyneuropathy (typically demyelinating) and a monoclonal plasma cell proliferative disorder (typically lambda light chain). One additional major criterion is required, including the presence of Castleman disease, sclerotic bone lesions, or an elevated VEGF. Finally, at least one minor criterion is required, including organomegaly, extravascular volume overload, endocrinopathy, skin changes, or thrombocytosis/polycythemia (Table 2) [3].

**Table 2:** Diagnostic Criteria for POEMS Syndrome [3] Mandatory criteria are always required for the diagnosis. At least one major criterion is required, as well as at least one minor criterion. Organomegaly includes hepatomegaly, splenomegaly, or lymphadenopathy. Volume overload can include peripheral edema, pleural effusion, ascites, or pulmonary edema. Endocrinopathy includes adrenal, pituitary, gonadal, parathyroid, or pancreatic abnormalities. Diabetes and thyroid abnormalities are not considered criteria due to their widespread prevalence. Skin changes include hyperpigmentation, hypertrichosis, glomeruloid hemangiomata, plethora, acrocyanosis, flushing, and white nails. This patient met both mandatory criteria and met the major criteria of elevated VEGF levels and the minor criteria of organomegaly as well as volume overload (peripheral edema). VEGF: vascular endothelial growth factor; POEMS: polyneuropathy, organomegaly, endocrinopathy, monoclonal gammopathy, and skin changes.

Mandatory criteria	Major criteria (at least one)	Minor criteria (at least one)
Polyneuropathy	Sclerotic skeletal lesions	Organomegaly
Monoclonal plasma cell disorder	Elevated VEGF levels	Volume overload
	Castleman disease	Endocrinopathy
		Skin changes
		Papilledema
		Thrombocytosis or polycythemia

Due to its rarity, there is no standard therapeutic strategy for POEMS. In fact, treatment regimens are largely based on prior retrospective studies, as well as extrapolated from treatment strategies in patients with multiple myeloma or AL amyloidosis. Fortunately, compared with multiple myeloma, the prognosis of POEMS is superior, with a median overall survival of 13.8 years, and this estimate is prior to the incorporation of many of the novel therapies employed today [4]. Generally, treatment is aimed at targeting the plasma cell clone along with upfront autologous stem cell transplant with high-dose melphalan conditioning for transplant-eligible patients. Autologous stem cell transplant leads to a durable response with five-year progression-free survival on the order of 74% and overall survival of 89% [7].

The immunomodulatory drug, lenalidomide, in combination with corticosteroids is the most studied initial induction regimen with a clinical and biochemical response rate of approximately 75% to 95% [8]. The proteasome inhibitor, bortezomib, is another option, yet treatment-related neuropathy remains a major concern given the often debilitating baseline neuropathy in many patients with POEMS. In comparison, incorporation of daratumumab, an anti-CD38 monoclonal antibody, represents a more promising therapeutic approach in combination with lenalidomide and dexamethasone, given its balance of tolerability and efficacy in both patients with multiple myeloma and POEMS. Multiple case reports have highlighted the encouraging upfront clinical and hematologic response rates when daratumumab is incorporated with lenalidomide and dexamethasone [9]. However, the lack of standardized clinical trials and drug cost have limited its use, as in our patient.

## Conclusions

POEMS is a systemic disease thought to be driven by a plasma cell clone that can result in multiple debilitating end-organ manifestations. Clinicians must maintain a high index of suspicion as early diagnosis and treatment are paramount. This highlights the importance of evaluating for secondary causes of peripheral neuropathy. Given the systemic nature of POEMS, treatment is aimed at targeting the plasma cell clone, though its rarity continues to make POEMS a diagnostic and therapeutic challenge.
